# Forage selection of dairy cows grazing contrasting multispecies swards is influenced by cow characteristics^☆^

**DOI:** 10.1016/j.vas.2026.100833

**Published:** 2026-09-02

**Authors:** Marie T. Dittmann, Mira Hesselmann, Geoffrey Mesbahi, Sarah Thorne, Amarante Vitra, Andrea K. Steiner, Florian Leiber

**Affiliations:** Department of Livestock Science, Research Institute of Organic Agriculture FiBL, Ackerstrasse 113, 5070 Frick, Switzerland

**Keywords:** Feeding behavior, Dietary choice, Botanical biodiversity, Plant secondary compound, Ruminant

## Abstract

Artificial pastures for dairy cows often comprise a small number of plant species, which limits the animals’ opportunity for forage selection. Despite being a natural behaviour observed in many ruminants, little is known about the factors influencing forage selection in domestic cattle. In this study, a herd of dairy cows was observed grazing on an experimental pasture, which was composed of four distinct swards of different botanical and chemical composition. The cows’ forage choices were recorded and it was tested whether there were statistical associations with cow characteristics, such as age, lactation status, days in milk (DIM), body condition score (BCS) or somatic cell count (SCC). The results revealed that with increasing age, cows consumed forage lower in grasses (P = 0.001), but higher in legumes (P = 0.002) and species diversity (P = 0.009). Lactating cows consumed forage higher in legumes (P = 0.007) and total extractable phenols (TEP, P = 0.009) than dry cows. With increasing DIM, cows consumed forage lower in grasses (P = 0.001) and higher in legumes (P = 0.002), TEP (P = 0.023) and condensed tannins (P = 0.029). With increasing SCC levels, cows consumed forage with less grasses (P = 0.017), a higher proportion of medicinal herbs (P = 0.030) and higher species diversity (P = 0.012). There were no associations between pasture choices and BCS. The results indicate that dairy cows show complex forage selection based on their physiology, age and health status. Giving cows the opportunity to select from a diverse array of pasture plants may increase their performance, health and wellbeing.

## 1 Introduction

In modern farming, most livestock species are kept in large groups and management systems are designed to match the overall requirement of the herd to optimize production. Stable measurements, diurnal rhythms, climatic conditions, or feed composition are all designed to meet the needs of the herd average (Rumphorst et al., 2022). However, research of the last decades has shown that different livestock species show strong individual variation in their needs and personalities (Adamczyk et al., 2023; Neave et al., 2018; Setser et al., 2023). Still, individuality is rarely considered in livestock husbandry, even when assessing animal welfare (Lundmark et al., 2015; Winckler, 2019). Instead of meeting their needs, “outliers” are often removed from the group or excluded from breeding, as catering for individual requirements is inefficient. However, consumers are increasingly critical towards industrial livestock farming and concerned about the welfare of farm animals (Alonso et al., 2020). Considering the individual, rather than the herd average, may become of increasing importance to improve animal welfare (Winckler, 2019).

One factor that is considered paramount for achieving high milk yields and optimal milk composition from dairy cows is the formulation of the ration (Tyasi et al., 2015). To meet the requirement of the average (lactating) cow, indoor diets are carefully formulated to avoid imbalances and deficiencies. To prevent selection and ensure the intake of all components at the desired concentration, the feed particles are reduced in size and mixed thoroughly (Schingoethe, 2017). These mixed rations are often complemented by pastures consisting of a limited number of plant species, e.g. clover and ryegrass. Bearing in mind that feed selection is a natural behavior in ruminants, providing cattle with the opportunity to express this behavior should be considered a crucial aspect of animal welfare (Leiber et al., 2020). Even though cows show a high steady intake of mixed rations, selection for certain components is still observed, even in total mixed rations (TMRs) (Schingoethe, 2017). Feed selection in ruminants is driven by factors such as the animal’s personality (Meagher et al., 2017; Neave et al., 2018), morphology and physiological status (Gregorini et al., 2008; Villalba & Provenza, 2007), nutritional state (Mangel & Clark, 1986; Scott & Provenza, 1999), ruminal microflora (Gregorini et al., 2008), breed (Dumont et al., 2007; Hessle et al., 2014), health status (Poli et al., 2018; Villalba et al., 2006), or previous experience (Lyman et al., 2011). Research has shown that when calves are allowed to select the diet components themselves, there are big individual differences which have no negative impact on the animals’ performance (Atwood et al., 2001).

In a previous study (Hesselmann et al., 2025), we found that a herd of dairy cows foraging on a pasture with spatially segregated multispecies swards on average spent the most time grazing in the swards rich in legumes. However, various individuals showed foraging patterns deviating from this herd average, spending considerable proportions of time grazing on swards rich in various forb species. The objective of this study was to understand if this variation was due to animal-related factors and if these are associated with individual forage selection. Specifically, we tested whether lactation status, somatic cell count, age and BCS were related to preferences for forage differing in botanical composition and concentrations of protein, tannins, and other secondary plant compounds. More broadly, the study sought to improve our understanding of how physiological and health-related cow characteristics may influence forage selection, thereby contributing to the development of more individualized approaches to dairy cow management.

The following specific hypotheses were investigated:

1) Due to an increased requirement for protein, lactating cows wereexpected to have a higher preference for swards rich in protein than dry cows. This hypothesis is supported by previous research, indicating that lactating cattle or sheep have a higher preference for forage rich in legumes (and thus protein) than non-lactating animals (Rutter, 2006).
2) In low dosages, tannins have been shown to improve the utilization of dietary protein (Min & Hart, 2003; Poutaraud et al., 2017). Therefore, lactating cows with higher protein requirements were expected to have a higher preference for forages rich in tannins.
3) Cows with increased somatic cell counts in their milk were expected to have a higher preference for forage containing herbs with potential medicinal effects like tannins and essential oils, which have been suggested to be effective in treating mastitis (Mushtaq et al., 2018).
4) There is evidence in mammals that preferences for different flavors change with age. For example, the sensitivity to bitter flavor changes with age in humans and mice (Mennella & Bobowski, 2015; Narukawa et al., 2017). Therefore, we expected a relationship between cow age and the preference for forage rich in polyphenolic compounds, which result in bitter taste (Soares et al., 2020).

In addition to testing these specific hypotheses, the data were explored for other relationships between cow characteristics and pasture properties.

## 2 Material and methods

### 2.1 Experimental site

The experimental site and the observational protocol are outlined and illustrated in detail in Hesselmann et al. (2025). In brief, a herd of 23 Swiss Fleckvieh dairy cows was observed grazing on an experimental pasture consisting of 16 plots with four different plant mixtures, located in Frick, Switzerland (47°30′51′′ N 8°1′26′′ E, 350 m a.s.l), divided into two pasture blocks of 8 plots each. All mixtures contained grasses and varying amounts of legumes and forbs. They were distinguished by the following properties: rich in grasses (*Festuca arundinacea* Schreb., *F. rubra* L., *Lolium perenne* L., *Poa pratensis* L.) (G), rich in grasses and legumes (*Trifolium* pratense L., *T. repens* L. and a small proportion *Medicago sativa* L.) (L), rich in grasses and plants that contain tannins (*Cichorium* sp. L.*, Lotus corniculatus* L.*, Plantago lanceolata* L.), and rich in grasses and herbs containing essential oils (*Achillea millefolium* L.*, Carum carvi* L. and at small proportions *Origanum vulgare* L. and *Thymus vulgaris* L.) (E). Other plant species, which had not been seeded occurred spontaneously, such as *Taraxacum officinale* aggr.. Pasture properties of the four mixes are shown in Table 1.

**Table 1:** Botanical and nutrient composition of the four plant mixtures on the experimental pasture, previously published in more detail Hesselmann et al. (2025). Values are averaged arithmetically for the four swards over six rotations (± SD). The DM yield is summed up over the entire grazing season from April to October.

	Essential oil	Grass	Legume	Tannin
Grasses %	31.5 ± 17.4	58.1 ± 21.3	26.4 ± 10.9	17.1 ± 13.2
Legumes %	46.3 ± 17.6	27.5 ± 18.9	68.5 ± 12.2	38.3 ± 15.1
Forbs %	22.3 ± 14.9	14.4 ± 13.1	5.1 ± 6.6	44.6 ± 13.6
Medicinal herbs %	20.7 ± 12.1	10.6 ± 7.8	3.4 ± 3.0	58.5 ± 12.6
Species diversity (n)	10.9 ± 2.2	6.5 ± 1.6	6.8 ± 1.4	10.8 ± 1.7
DM yield (t ha^-1^)	7.93 ± 0.61	8.00 ± 0.72	9.04 ± 0.41	6.95 ± 0.37
CP (g 100 *g*^-1^)	19.1 ± 2.0	17.9 ± 1.7	21.7 ± 0.7	17.6 ± 1.4
NEL (MJ kg^-1^)	6.1 ± 0.2	5.9 ± 0.2	6.3 ± 0.4	5.8 ± 0.2
TEP (g 100 *g*^-1^)	1.95 ± 0.03	1.94 ± 0.08	2.02 ± 0.08	2.31 ± 0.16
CT (g 100 *g*^-1^)	0.45 ± 0.05	0.32 ± 0.02	0.38 ± 0.02	0.89 ± 0.26

DM = dry matter, CP = crude protein, NEL = net energy lactation, TEP = total extractable phenols, CT = condensed tannins.

### 2.2 Study animals

The dairy herd observed in this study comprised 23 cows and heifers housed on a commercial farm. The sample size was limited by the number of cows in the herd. The cows’ age ranged between 2 and 8 years (mean: 4). Over the grazing season, lactation numbers ranged from 0 to 5 and the cows’ BCS (determined based on Isensee et al., 2014) from 2.75 to 3.75 (mean: 3.25). At the start of the observations the herd included three heifers, all of which entered their first lactation during the study. Before calving they were classified as dry cows. During the study, three cows changed their status from dry to lactating, while 9 changed their status from lactating to dry. While stabled, cows received hay ad libitum and small amounts of grass-clover silage. Lactating cows additionally received limited quantities of a wheat-bran-based pelleted parlor feed. The average milk yield recorded by the milking robot (Lely Astronaut) amounted to 12 kg/d ± 4.6 with a range of 3–19 kg/d. Newborn calves were allowed to drink from their mothers for at least two weeks after birth and recorded milk yield does not account for this loss. Therefore, this variable was not included in statistical analysis.

### 2.3 Behavioral observations and grazing management

The foraging behavior of the herd on the experimental pasture was observed during 6 grazing rotations, each consisting of 6 to 8 consecutive days, during which the cows grazed for 1.5 to 6 h on the experimental pasture. The observations consisted of scan sampling at 10-minute intervals, recording the location (defined as presence in a certain plot during observation) and foraging behavior of each cow (feeding, ruminating, or non-ingestive behaviors). While on pasture, cows could move freely within one of the two pasture blocks, which comprised 8 plots each, i.e. two plots of each mixture. The blocks were grazed at daily alternation during each grazing cycle. To ensure that the cows always had the option to graze on all mixtures, plots were not grazed below an average rising plate meter (RPM) height of 10. RPM measurements were taken after each day the cows had been observed on the experimental pasture. After each grazing rotation, all plots were topped to 8 cm to ensure an even regrowth of the vegetation. The pasture was then rested between 2.5 and 4 weeks before the next grazing cycle. During this time, the herd grazed on other pastures and was not observed.

### 2.4 Botanical surveys

Two days before each grazing cycle, the botanical composition of each plot was assessed by sampling six 0.25 m^2^ quadrats per plot, based on Klapp (1965). Species diversity was defined as the number of dicotyledonous species occurring within a quadrat. As the botanical surveys disregarded the presence of different grass species, these were not included in the species diversity variable. Based on the botanical surveys, a variable was created to approximate the proportion of herbs with potential medicinal benefits. The species included in this variable (termed ‘medicinal herbs’) are known for their use in veterinary phytotherapy, generally due to their high concentrations in plant secondary metabolites (PSM), such a polyphenols or essential oils (Reichling et al., 2016; Wynn & Fougère, 2006): *Achillea millefolium* L., *Artemisia absinthium* L., *Carum carvi* L., *Cichorium* sp., *Hypericum perforatum* L., *Lotus corniculatus* L., *Origanum vulgare* L., *Plantago lanceolata* L., *Sanguisorba minor* Scop., *Taraxacum officinale* aggr., *Thymus vulgaris* L..

### 2.5 Forage analysis

Pasture samples were taken of the quadrats and dry matter (DM) was determined by oven drying for 24 h at 60 °C. After milling the samples to a particle size of 1 mm, nutrient analyses for organic matter (OM), crude protein (CP), acid detergent fiber (ADF), acid detergent lignin (ADL), neutral detergent fiber (NDF) and crude fat (CF) contents were performed by an external laboratory, according to European standard methods (Bundesamt für Verbraucherschutz und Lebensmittelsicherheit, 2025). Total extractable phenols (TEP) and condensed tannin (CT) contents were determined as described in Kapp-Bitter et al. (2023). Net energy for lactation (NEL) was calculated according to Daccord et al. (2017). The results of the botanical survey and the nutrient analysis are shown in Table 1.

## 3 Data analysis

The relationships between the animal characteristics and the properties of the pasture they chose to graze on were assessed by the aid of linear mixed models. The original dataset was based on animal observations, containing information on which plot a cow was observed foraging on for each scan of each rotation (n = 14′931 scans). This dataset was extended by adding the botanical and nutritive properties for the chosen plot at the time of the scan, based on the botanical survey and the chemical analysis. Botanical data were based on a dataset of average values per plot per rotation (n = 96), which included the proportion of grasses, legumes and forbs, NEL as a proxy for energy density, CP content, TEP content as a proxy for plant secondary metabolites, which may have medicinal effects or cause a bitter taste, CT content as a proxy for tannin content, species diversity and the content of plants with potential medicinal benefits.

Furthermore, the dataset was extended by adding information on the cow during the time of the scan: Age, DIM, BCS (scoring system with 0.25-point increments), lactation status (lactating or dry), SCC (if the cow was lactating). Like for the nutrient values, numerical values were averaged per rotation. The final dataset was then condensed by creating an average per cow per rotation to avoid bias due to different numbers of observations during the six rotations. Different numbers of observations were caused by missing values due to cows calving and not being on pasture, or different numbers of scans due to variation in daily grazing duration and the number of days the cows were observed during one grazing cycle. This resulted in a dataset of 138 averages per cow per rotation, on which the linear mixed models were applied. The pasture variables of the chosen forage were included as outcome variables in the mixed models. Cow variables at each rotation were included as fixed factors in separate univariate models: BCS, lactation stage (lactating or dry), DIM (days in milk, which was deemed 0 for heifers and dry cows), and age (which was set to a fixed value for each cow throughout all rotations). Somatic cell count (SCC) was based on data automatically recorded by the milking robot (Lely Astronaut) and was therefore only available for lactating cows. Due to the non-normal distribution of SCC values, this variable was log-transformed for analysis.

Each model was used to test one hypothesis by including one outcome variable (pasture properties), one fixed factor (cow properties), as well as Cow ID as a random factor. Cow variables and pasture properties were tested separately for cross-correlation using Pearson or Spearman correlations, depending on the variables. Furthermore, cow variables were tested for correlation with the number of grazing rotations, to detect confoundment due to temporal variation in the pasture properties. For a better visual understanding of the associations between cow characteristics and pasture variables, variable factor maps were created based on principal component analysis (PCA) of all variables of interest. R Studio (R version 1.4.1717) was used to perform all statistical analyses and to create illustrations, using the packages lmerTest, psych, plyr, FactoMineR and factoextra.

## 4 Results

The descriptive results on foraging preferences of the individual cows throughout the five grazing rotations are provided and illustrated in detail in Hesselmann et al. (2025).

### 4.1 Associations between cow characteristics and pasture properties

Based on the linear mixed models there were several statistically significant relationships between cow characteristics and the average composition of the forage they consumed while grazing on the experimental pasture. In the following, the statistically significant associations between cow characteristics and pasture properties are summarized, while all effect sizes and p-values are listed in Table 2.

**Table 2:** Results of the linear mixed models investigating the relationship between cow characteristics and pasture variables. The numbers indicate the fixed effect and the P-value of the mixed models. In the case of the binary variable lactation status, the fixed effect refers to the difference between the two categories (lactating / dry), where the reference level is lactating.

		Cow characteristics / fixed effects				
Pasture variables / outcome variables		Age (y)	Lactation status (lactating / dry)	DIM (d)	BCS (scoring points)	SCC (log)
Grasses (%-point)	Effect	−1.10	−2.38	−0.02	−0.09	−1.35
	P-value	0.001	0.219	0.004	0.965	0.017
Legumes (%-point)	Effect	0.87	4.25	0.02	−1.28	0.63
	P-value	0.002	0.007	0.002	0.468	0.204
Forbs (%-point)	Effect	0.24	−1.74	0.002	1.39	0.72
	P-value	0.381	0.261	0.638	0.404	0.116
Medicinal herbs (%-point)	Effect	0.49	−3.27	0.0002	1.06	1.18
	P-value	0.138	0.08	0.974	0.603	0.030
Species diversity (n)	Effect	0.10	0.25	0.0001	−0.43	0.16
	P-value	0.009	0.266	0.921	0.077	0.012
Yield (t ha^-1^)	Effect	−0.003	0.03	−0.0002	−0.03	−0.02
	P-value	0.773	0.653	0.376	0.603	0.347
NEL (MJ kg^-1^)	Effect	0.005	0.09	0.0001	0.01	−0.008
	P-value	0.598	0.085	0.348	0.833	0.592
CP (g 100 *g*^-1^)	Effect	0.10	0.43	0.001	−0.54	0.08
	P-value	0.095	0.185	0.373	0.125	0.411
TEP (g 100 *g*^-1^)	Effect	0.01	1.34	0.005	0.58	−0.05
	P-value	0.904	0.023	0.009	0.360	0.764
CT (g 100 *g*^-1^)	Effect	0.06	0.235	0.005	0.94	0.15
	P-value	0.651	0.613	0.029	0.210	0.469

DIM = days in milk, BCS = body condition score, SCC = somatic cell count, NEL = net energy lactation, CP = crude protein, TEP = total extractable phenolics, CT = condensed tannins.

For each additional year of age, the percentage of grasses in the forage the cows consumed decreased by 1.1% (P = 0.001) and the percentage of legumes increased by 0.87% (P = 0.002). For each additional year of age, species diversity in the forage increased by 0.1 species (P = 0.009) and the CP content tended to increase by 0.1 g per 100 g DM forage (P = 0.095).

On average, the forage consumed by lactating cows contained 4.25% more legumes (P = 0.007) and 1.34 g more TEP per 100 g DM forage (P = 0.023) than that of dry cows. There were trends that the forage consumed by lactating cows contained 3.3% less medicinal herb species (P = 0.08) and had a 0.09 MJ per kg DM higher NEL value (P = 0.09) than that consumed by dry cows.

For each additional DIM, the percentage of grasses in the consumed forage decreased by 0.02% (P = 0.004) and the percentage of legumes in the forage increased by 0.02% (P = 0.002), as did the content of TEP by 0.005 g per 100 g DM forage (P = 0.009) and the content of CT by 0.005 g per 100 g DM forage (P = 0.029).

BCS showed no statistically significant associations with any of the pasture properties, but there was a trend that with each additional BCS point, the number of species in the consumed forage decreased by 0.4 (P = 0.077). With increasing SCC, the forage consumed by the cows contained more medicinal herbs (P = 0.030), had a higher species diversity (P = 0.012) and a lower percentage of grasses (P = 0.017). In summary, of the initial hypotheses, only hypothesis 3 could be confirmed.

### 4.2 Correlations between variables

For the interpretation of the mixed model results, the correlation between cow characteristics and between pasture variables must be considered. The cross-correlation matrix for cow characteristics is shown in Table 3. Age was positively correlated with SCC. BCS was higher in lactating than in dry cows. Lactating cows had higher DIM than dry cows, which is caused by the definition of dry cows being at 0 DIM. None of the cow characteristics were correlated with rotation number, indicating that none of the cow variables showed consistent temporal progress, which may have been confounding with a seasonal progress in pasture properties.

**Table 3:** Cross-correlation of cow characteristics. Values in the top right cells contain P-values, values in the bottom left cells contain Rho. Correlations are based on average values per cow per rotation (n = 138). Rotation number is a consecutive number of the six grazing rotations and is used as a proxy for seasonal progress.

	Cow age	DIM	SCC	BCS	Lactating (/dry)	Rotation number
Cow age	-	0.207	<0.001	0.907	0.807	0.932
DIM	0.11	-	0.533	0.138	<0.001	0.198
SCC	0.42	0.06	-	0.535	NA	0.696
BCS	0.00	−0.03	−0.02	-	0.004	0.251
Lactating (/dry)	−0.03	−0.88	NA	0.04	-	0.182
Rotation number	0.00	−0.11	0.04	−0.09	−0.11	-

DIM = days in milk, SCC = somatic cell count, BCS = body condition score.

There were various statistically significant correlations between pasture properties (Table 4). The proportion of grasses was negatively correlated with the content of legumes, forbs, medicinal herbs, CP, TEP, CT, and species diversity. Legume content was negatively correlated with the content of forbs and medicinal herbs, and positively correlated with the content of CP and NEL. The content of forbs was positively correlated with TEP and TT, and negatively with yield, CP and NEL. Furthermore, it was positively correlated to species diversity and the content of medicinal herbs, which is due to all medicinal herbs being forbs and species diversity being only assessed for dicotyledonous plants. For the same reason, the content of medicinal herbs was positively correlated with species diversity. Furthermore, the content of medicinal herbs was positively correlated with TEP and CT, and negatively correlated with yield, CP and NEL. Yield was positively correlated with NEL. NEL was negatively correlated with CT. TEP was positively correlated with CT.

**Table 4:** Cross-correlation of pasture properties. Values in the top right cells contain P-values, values in the bottom left cells contain Rho. Correlations are based on average values per plot per rotation (n = 96).

	Grasses %	Legumes %	Forbs %	Medicinal herbs %	Species diversity	Yield (t ha-1)	CP (g/100 g)	NEL (MJ/kg)	TEP	CT
Grasses %	-	<0.001	<0.001	<0.001	<0.001	0.113	<0.001	0.963	0.002	<0.001
Legumes %	−0.59	-	<0.001	0.004	0.789	0.105	<0.001	<0.001	0.427	0.421
Forbs %	−0.50	−0.41	-	<0.001	<0.001	<0.001	0.003	<0.001	0.009	<0.001
Medicinal herbs %	−0.54	−0.29	0.92	-	<0.001	0.007	0.001	<0.001	0.001	<0.001
Species diversity	−0.40	0.03	0.43	0.45	-	0.231	0.273	0.405	0.090	0.112
Yield (t ha^-1^)	0.16	0.17	−0.36	−0.27	−0.12	-	0.854	0.001	0.486	0.397
CP (g 100 *g*^-1^)	−0.39	0.69	−0.30	−0.33	−0.11	−0.02	-	<0.001	0.819	0.342
NEL (MJ kg^-1^)	0.00	0.50	−0.55	−0.56	−0.09	0.33	0.44	-	0.090	0.001
TEP (g.100 *g*^-1^)	−0.32	0.08	0.27	0.32	0.17	−0.07	−0.02	0.17	-	<0.001
CT (g 100 *g*^-1^)	−0.42	0.08	0.39	0.58	0.16	−0.09	−0.10	−0.34	0.53	-

NEL = net energy lactation, CP = crude protein, TEP = total extractable phenolics, CT = condensed tannins.

### 4.3 PCA and variable factor maps

**Fig. 1:**
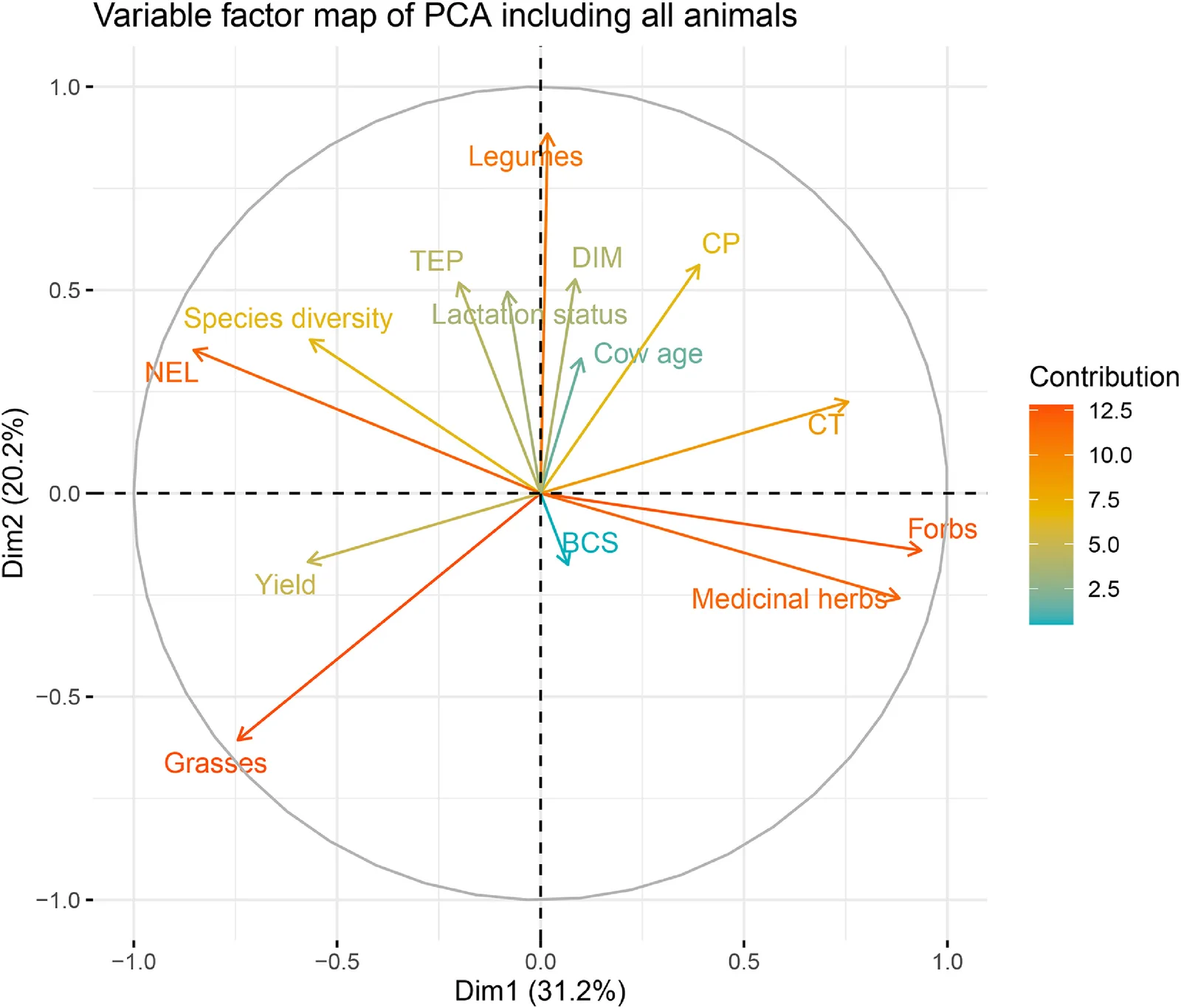

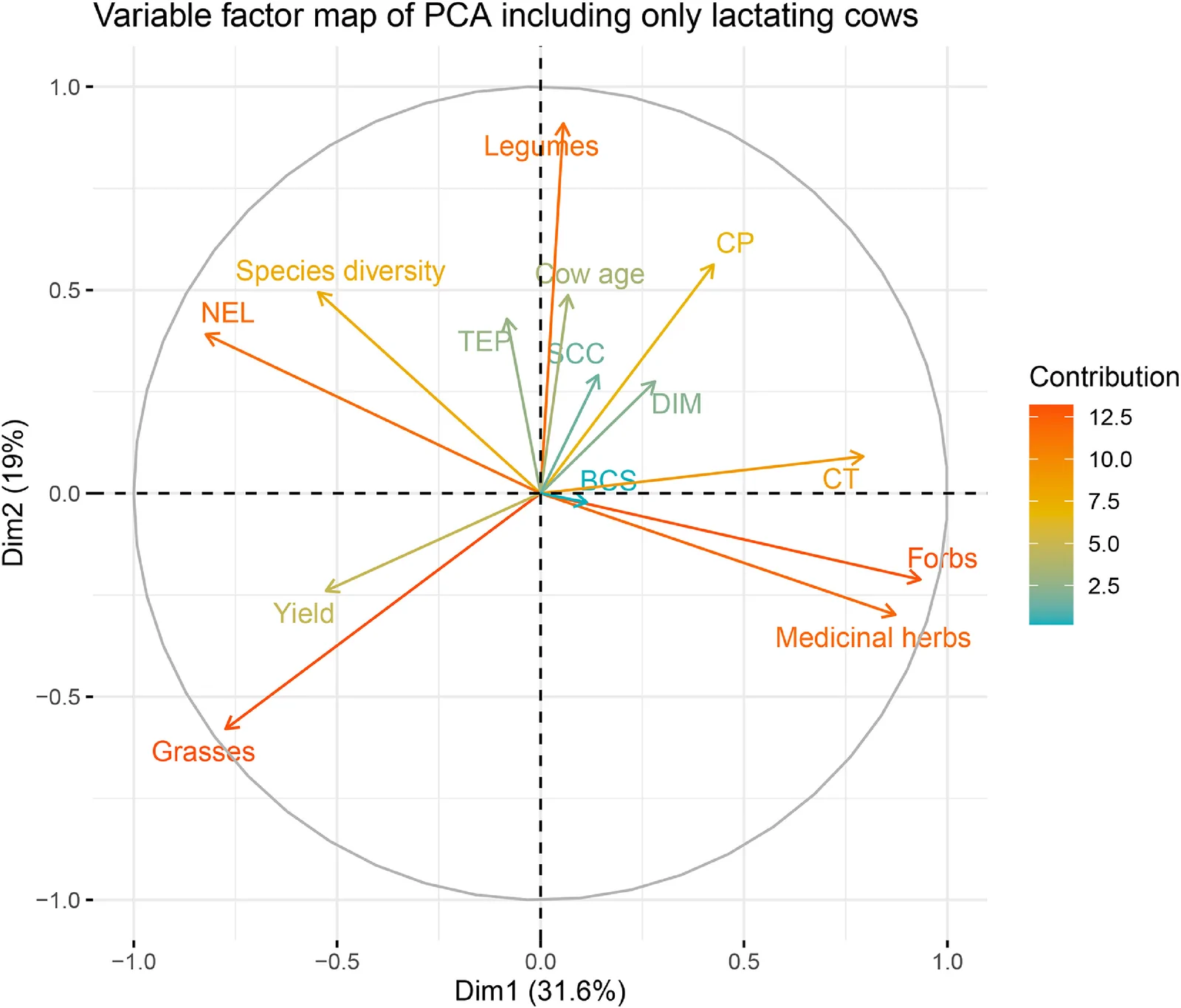
A) Variable factor map based on data of all animals, excluding the variable SCC due to missing values in dry cows. For the binary variable Lactation status, lactating was classified as 1 and dry as 0. B) Variable factor map for data from lactating cows only, including the variable SCC. Percentages in brackets indicate how much variance the principal components Dim1 and Dim2 explain in the dataset. Color and length of the arrows indicate the contribution of the variable to either Dim.

Fig. 1 illustrates the results of the PCA performed on cow characteristics and pasture properties on two datasets: including all animals but no SCC values (due to missing values for dry cows, Fig. 1A) and one including data from lactating cows only, therefore including the variable SCC (Fig. 1B). Overall, the figures match the results of the univariate mixed models presented in Table 2.

## 5 Discussion

### 5.1 Study limitations

Before discussing the results, it should be noted that there were some limitations to the design of this study. The data originate from only one dairy herd, including 23 female animals of one breed, grazing on one pasture. While observations were carried out on different days and seasons, there was no replication with different animals or breeds. It should further be noted that the findings are correlative rather than causative, and that correlations between cow characteristics and pasture properties should be considered when interpreting the results.

### 5.2 Discussion of the results

When tailoring diets to meet the needs of dairy herds, some production systems already aim at adapting nutrient intake to the different stages of lactation. Some automated milking systems (AMS) allow the individual feeding of concentrates (Henriksen et al., 2019), which is a step towards tailoring rations to animal-specific requirements, but purely with the target of increasing feed conversion and yields. However, the cows’ choices would naturally be based on various metabolic needs such as rumen process balancing, protein and fatty acid protection, toxin inhibition, and curation of parasitic diseases. There is sound evidence that ruminants manage these challenges by making selective choices from arrays of different plants with different contents of nutrients and phytochemicals (Atwood et al., 2001; Lyman et al., 2011; Min & Hart, 2003; Poli et al., 2018; Rutter, 2006; Villalba et al., 2006). In agricultural reality, their choices are often limited to total mixed rations of pelleted concentrates and forage consisting mainly of grasses or clover. With this limited number of items on the menu, dairy cows lack the option for true feed selection, which may bring along not only metabolic shortcomings but also impaired wellbeing (Leiber et al., 2020).

The results of this study suggest that dairy cows show considerable individuality in forage selection, influenced by physiological traits such as age, lactation stage, or SCC.

Regarding the initial hypotheses, only one could be confirmed: There was a positive relationship between SCC and the proportion of medicinal herbs in the selected forage. There is evidence supporting the use of phytotherapy in the treatment of mastitis (Mushtaq et al., 2018; Walkenhorst et al., 2020). Although this finding should be interpreted tentatively, it may be an indicator that cows seek out plants rich in PSM when their health is challenged. Future experimental work may clarify whether cows actually practice self-medication when suffering from mastitis.

Several other interesting associations between cow characteristics and pasture properties were found. With increasing age, animals consumed forage lower in grasses, higher in legumes and higher in species diversity. Some sources suggest that the digestive efficiency of certain nutrients declines with age (National Research Council, 2001). The consumption of more protein by older cows may therefore represent a mechanism to optimize carbohydrate digestion, as the balance between protein and carbohydrates in the rumen is crucial for microbial digestion (Nocek & Russell, 1988). With increasing cow age, the CP content in the forage tended to increase. This is in disagreement with the theory that younger, growing animals have a higher requirement for protein than older animals (National Research Council 2001). However, the youngest animals in this study were around two years old, by which age most of the physical growth is completed and the protein requirement for synthesis of muscle and organ tissue should decline. Another relevant explanation for a lower intake of legumes and CP in younger animals may be herd hierarchy (Neave et al., 2018). Older animals may show dominance towards younger cows and heifers, preventing them from accessing high-quality forage, thereby forcing them to consume forage lower in CP and higher in grasses.

The increased preference for legumes and forages richer in plant diversity with increasing age may also be caused by a shift in preference. In humans and rodents, it has been found that taste perception changes with age. For example, with increasing age, people perceive tastes as less intense (Barragán et al., 2018), and the sensitivity for bitter flavours decreases with increasing age in humans and mice (Mennella & Bobowski, 2015; Narukawa et al., 2017). Whether similar age-related changes occur in cattle remains unclear, but altered sensory perception may represent one possible explanation for the observed differences in forage selection, i.e. that older cows investigated in this study were less sensitive to the possibly stronger flavors of the legumes and other dicot species. In addition to taste perception, an increased intake of forage with more legumes may be a learned behavior (Villalba & Provenza, 2009). Older cows may have had more time to experience the – potentially positive – effects of legume-rich forage, which may have resulted in them consuming it more frequently than younger animals. However, older cows did not show a higher consumption of presumably bitter forage high in TEP, CT or medicinal herbs.

Lactating cows consumed forage higher in legumes and TEP than dry cows and they tended to consume forage with higher NEL values and less medicinal herbs than dry cows. Interestingly, the CP content in their diet was not significantly elevated compared to dry cows (although there was a numerical increase). Increased requirements in protein and energy in lactating cows can be explained by milk production. The higher content of TEP in the forage consumed by lactating cows and with increasing DIM may be due to certain health benefits of phenolic compounds. Research suggests that phenolics may reduce the negative effects of oxidative stress (Formato et al., 2022), thereby supporting animal health. Considering that oxidative stress in cows increases with parturition and the onset of lactation (Sharma et al., 2011), an increased consumption of forage rich in TEP may be a mechanism to reduce oxidative stress.

With increasing DIM, cows consumed forage lower in grasses but higher in legumes, and higher in CT. Although there were no significant associations between DIM and CP or NEL, a shift of from a grass-rich diet to a legume-rich diet may mirror different nutrient requirements at different lactation and gestation stages. Tannins protect polyunsaturated fatty acids from rumen biohydrogenation (Khiaosa-Ard et al., 2009; Willems et al., 2014). These fatty acids, in particular n-3 fatty acids, are essentially important for embryo development (Sinclair et al., 2002). A gravid female ruminant could exhibit feeding preferences aimed at increasing the supply of n-3 fatty acids to the developing fetus (Burdge et al., 2003).

BCS did not show significant associations with any of the pasture choices. The only finding was that there tended to be a negative association between BCS and the proportion of medicinal herbs in the chosen forage. The variation of BCS in the investigated herd may have been too limited to detect associations with this variable. Moreover, BCS may be too strongly confounded with milk yield and gestation to reveal distinct effects on feeding choices.

There was some discrepancy between the relationships among pasture properties in the botanical dataset and those observed in the dataset based on the cows' feeding locations. For example, there were positive relationships of the proportion of forbs with species diversity and TEP content in the botanical dataset. However, in the variable factor maps based on the scan sampling dataset, the angles between the arrows of these variables indicate negative relationships of forb proportion with TEP content and species diversity. This finding could indicate that the cows that seek out forb dominated plots may feed in plots that are high in forbs but low in species diversity (e.g. plots dominated by L. *corniculatus* or *Cichorium*), or, vice versa, high in legumes and grasses and species rich.

Although this study focused on dairy cows, the concept of linking individual forage selection to physiological status may also be relevant in other ruminants. Beef cattle, sheep and goats likewise face changing nutritional and health-related demands associated with growth, reproduction, lactation, parasitic challenge, and disease. The ability to select among plant species differing in nutrient composition and phytochemical content may therefore represent a general adaptive strategy across grazing herbivores. Investigating whether similar relationships between physiological traits and forage choice occur in other livestock species could improve our understanding of animal-driven diet selection and support the development of grazing systems providing livestock with opportunities for forage selection.

In summary, the data revealed several associations between the physiological status of dairy cows and their choice of different forage compositions on pasture. The results of this study contribute to an understanding of cows as individual decision-makers with unique needs and preferences that may not always be represented when tailoring husbandry systems for the herd average. Providing cows with the opportunity to graze on multispecies swards enables them to express those individual preferences and may help them better match nutrient and phytochemical intake to their physiological state. From a practical perspective, these findings support the development of grazing systems that maintain botanical diversity and offer animals greater opportunities for dietary choice. Such systems may not only promote animal welfare but could also improve the ability of animals to cope with changing nutritional requirements and health challenges throughout lactation and life. While the findings of this study should be interpreted in light of the study limitations, they provide hypotheses for future experimental studies and suggest that monitoring grazing choices may offer useful information about the physiological needs and health status of individual animals. Further research is needed to determine whether offering dairy cows a diverse array of pasture plants enhances their performance, health, and well-being, and whether similar benefits can be achieved in other grazing livestock systems.

## Ethics declaration

The experimental protocol was approved by the Animal Health and Welfare commission of the Canton Aargau, Switzerland (National license Nr. 34390).

## Funding

We gratefully acknowledge the generous financial support of the Foundation Edith Maryon, Basel.

## CRediT authorship contribution statement

**Marie T. Dittmann:** Writing – review & editing, Writing – original draft, Visualization, Supervision, Project administration, Methodology, Investigation, Formal analysis, Data curation, Conceptualization. **Mira Hesselmann:** Writing – review & editing, Writing – original draft, Methodology, Investigation, Formal analysis, Data curation. **Geoffrey Mesbahi:** Writing – review & editing, Investigation, Formal analysis. **Sarah Thorne:** Writing – review & editing, Methodology, Investigation. **Amarante Vitra:** Writing – review & editing, Methodology, Investigation, Formal analysis, Data curation, Conceptualization. **Andrea K. Steiner:** Writing – review & editing, Methodology, Investigation, Data curation. **Florian Leiber:** Writing – review & editing, Supervision, Funding acquisition, Conceptualization.

## Declaration of competing interest

None.
